# Influence of Live Music and Tasting Assessment on Hedonic and Emotional Responses of Wine in Public Tasting Events †

**DOI:** 10.3390/foods15030504

**Published:** 2026-02-01

**Authors:** Roberto Marangoni, Isabella Taglieri, Alessandro Bianchi, Chiara Sanmartin, Pierina Díaz-Guerrero, Alessandro Tonacci, Francesco Sansone, Francesca Venturi

**Affiliations:** 1 Department of Biology, University of Pisa, Via Luca Ghini 13, 56126 Pisa, Italy; roberto.marangoni@unipi.it; 2 Interdepartmental Center for Complex Systems Studies, University of Pisa, L.go B. Pontecorvo 3, 56127 Pisa, Italy; francesca.venturi@unipi.it; 3 Department of Agriculture, Food and Environment, University of Pisa, Via del Borghetto 80, 56124 Pisa, Italy; isabella.taglieri@unipi.it (I.T.); chiara.sanmartin@unipi.it (C.S.); pierina.guerrero@phd.unipi.it (P.D.-G.); 4 Interdepartmental Research Centre “Nutraceuticals and Food for Health”, University of Pisa, Via del Borghetto 80, 56124 Pisa, Italy; 5 Department of Pharmacy, University of Pisa, Via Bonanno Pisano 6, 56126 Pisa, Italy; 6 Institute of Clinical Physiology, National Research Council (IFC-CNR), Via Giuseppe Moruzzi 1, 56124 Pisa, Italy; alessandro.tonacci@cnr.it (A.T.); francesco.sansone@cnr.it (F.S.)

**Keywords:** wine sensory evaluation, emotional responses, hedonic liking, consumer experience, music-induced modulation, crossmodal perception

## Abstract

Wine represents one of the most complex food matrices from a sensory perspective, as its appreciation emerges from the interaction between chemical composition, perceptual mechanisms, and contextual influences. Contemporary research in oenology and sensory science increasingly recognizes wine evaluation as an integrated perceptual event shaped by cognition, memory, and affect, rather than a simple response to aroma or flavor cues. Live music is widely used in hospitality settings to enhance consumer experience; however, its specific influence on wine appreciation and emotional responses remains insufficiently explored, particularly in real-world contexts. This study investigates how two contrasting musical atmospheres—melancholic/relaxing and upbeat/motivational—modulate hedonic evaluations and emotional profiles during public wine tastings, compared with a no-music condition. Data were collected across five live tasting events (*5 Wednesdays of Emotions*) using structured questionnaires that included hedonic ratings and multidimensional emotional measures. Statistical analyses were conducted using non-parametric tests, meta-analytic *p*-value combination, and cumulative link mixed models for ordinal data. The presence of music significantly enhanced overall wine appreciation compared to the silent condition, although the magnitude and direction of the effect varied across individuals and musical styles. Upbeat/motivational music generally produced stronger and more consistent increases in liking than melancholic/relaxing music. Emotional responses—particularly positive surprise—emerged as key mediators of hedonic improvement and showed strong associations with overall liking. Preference profiling revealed distinct response patterns, indicating that auditory modulation of wine perception is not uniform across consumers. These findings support a crossmodal interpretation in which music shapes wine appreciation primarily through emotion-based and expectancy-related mechanisms rather than through direct sensory enhancement. By demonstrating these effects in ecologically valid tasting environments, the study highlights the role of auditory context as a meaningful component of multisensory wine experiences.

## 1. Introduction

Wine represents one of the most complex food matrices from a sensory perspective, and its appreciation arises from the interplay between chemical composition, perceptual mechanisms, and contextual influences [1,2,3,4]. The sensory perception of wine—including olfactory and tactile attributes—can be described as a multifaceted process driven by the physicochemical properties of each compound, matrix-mediated interactions between volatile and non-volatile substances, and the intrinsically subjective nature of human sensory experience [5,6,7,8,9,10]. In recent years, research in oenology and sensory science has increasingly emphasized that wine evaluation is not merely a matter of detecting aromas or tasting flavors, but rather the outcome of an integrated perceptual event shaped by cognition, memory, and affect. This view aligns with contemporary perspectives in affective neuroscience and crossmodal perception [11,12,13,14].

Emotions may be defined as psychophysiological responses elicited by the perception of salient stimuli, involving autonomic and endocrine changes, cognitive appraisal mechanisms, and—in some cases—activation through mnemonic recall or mental imagery [15,16,17]. Evolutionary evidence [18,19] suggests that valence-tagged sensory representations in emotion-related brain regions are not secondary features developed merely to support a centralized affective system. Instead, they reflect the primordial functions of these structures—remnants of an earlier stage in evolution when sensory experiences and emotional–motivational states were essentially inseparable. Such a perspective provides a useful framework for understanding why complex stimuli such as wine can trigger rich emotional impressions that go beyond basic liking or disliking [20,21,22].

Although the underlying mechanisms remain only partially understood, growing evidence indicates that interactions between cortical and thalamic areas are central to cognitive processing. Several lines of research now propose that a core feature of thalamic function is its ability to merge perceptual, emotional, and cognitive inputs into a unified, meaningful experience [23]. Furthermore, Taglieri et al. (2025) [2] demonstrated that the emotions measured were strongly correlated with quantitative and hedonic attributes obtained using classic sensory analysis, both by trained panelists and non-expert consumers, highlighting the relevance of emotional measurement in oenological studies.

Among all sensory modalities, olfaction most closely mirrors emotional processing in its assignment of positive (appetitive) or negative (aversive) valence to environmental cues. The tight anatomical connections between neural systems involved in olfactory processing and those supporting emotional functions [24] account for the strong and well-documented associations between these domains [25,26,27,28]. Given that wine evaluation relies heavily on olfactory information, emotional mechanisms are likely to contribute significantly to individual differences in preference, memory, and contextual sensitivity.

Crossmodal interactions between sensory modalities—such as auditory and gustatory channels—also play a crucial role in shaping consumer perception of food and beverages [13]. A growing body of research [29,30,31,32,33] demonstrates that music can influence attributes such as sweetness perception, emotional responses, and overall liking. However, empirical studies conducted in ecologically valid, real-consumer environments remain limited, particularly for complex products such as wine, where expectations, expertise, and contextual cues strongly modulate evaluation. As previously reported [31], understanding how auditory cues shape wine perception is therefore essential for both scientific and applied domains, including hospitality, marketing, and experience design.

In the wider frame of the research project “Cantina 5.0” [34], the present study investigates how musical atmospheres influence hedonic wine evaluations and emotional profiles during five distinct events belonging to a public tasting series titled “*5 Wednesdays of Emotions.*” Each event included a blind tasting of four different wines (one of which remained constant throughout the five events, to give us a standard reference point). The music was performed live by a jazz trio, whose members varied from one event to the next. For each wine, two songs were selected from a very wide jazz catalogue, to evoke contrasting emotional states: a melancholic/relaxing song (mel) and an upbeat/motivational song (upb). The order by which mel and upb songs were played was random for each wine, to avoid a drift in the music-induced emotions. Spectators were asked to complete an online survey in which they evaluated several emotional variables and provided an overall hedonic judgment. The latter was recorded three times: first in the absence of music, then while listening to the first song, and finally during the second song. We analyzed whether music modulates liking, whether the magnitude of this modulation differs between songs, and how emotional responses relate to hedonic outcomes. We further tested potential confounders such as tasting order, wine identity, and individual traits (gender, age, body type, wine experience, music experience).

This work aims at contributing to sensory science by (i) quantifying music-induced hedonic shifts in an ecologically valid setting, (ii) characterizing emotional correlates of liking, and (iii) assessing inter-individual variability using robust statistical methods, including non-parametric tests, meta-analytic combination, and cumulative link mixed models. By integrating emotional neuroscience, crossmodal perception, and consumer sensory science, the present study aims to advance our understanding of how multisensory environments shape real-world wine appreciation.

## 2. Materials and Methods

### 2.1. Study Design and Participants

#### 2.1.1. 5 Wednesdays of Emotions

The dataset derives from a public tasting series entitled “*5 Wednesdays of Emotions*”. Five live events were conducted, during which a variable number of attendees (ranging from 45 to 50 individuals) participated in evening sessions where wine tasting was either paired with or presented without accompanying jazz music (Figure S1). At each event participants completed a structured questionnaire via an online survey. Participants were recruited on a voluntary basis and anonymously among event attendees, with no incentives provided. Questionnaires with incomplete responses for key variables were excluded from specific analyses on a case-by-case basis; no data imputation was performed. The overall sample comprises repeated cross-sections across events; only three participants attended all five events, and five participants attended two events. Given this limited overlap, longitudinal dependency between events was considered negligible and each event was treated as largely independent for the main analyses. The evaluations were carried out with respect to ethical standards regarding human subject involvement, following health and safety protocols. The study was approved by the Ethical Clearance of CNR (Protocol no. 0291041, 21 August 2024).

#### 2.1.2. Wine Used in the Study

The 16 wines derive from commercial wineries in Italy (Friuli Venezia Giulia and Tuscany regions) collected within the project “Cantina 5.0”. The wine codes and associated information are reported in Table 1, and the chemical and color parameters of the wines are reported in Table S1.

### 2.2. Chemical Characterization of Wine

The wines were analyzed in triplicate by a WineScan FT 120 (Foss Analytics, Hillerod, Denmark) to quantify the following chemical parameters: alcohol (% *v*/*v*), sugars (g/L hexoses), pH, titratable acidity (g tartaric acid/L), volatile acidity (g/L acetic acid), malic acid (g/L), lactic acid (g/L), glycerol (g/L), total extract (g/L), total anthocyanins (mg malvidin/L), total polyphenols (mg gallic acid/L). The accuracy of the Fourier transform infrared WineScan measurements was validated by destructive analyses conducted according to OIV methodologies, as previously outlined [35,36].

The color of wine was assessed by means of a tristimulus colorimeter (Eoptis, Mod. CLM-196 Benchtop, Trento, Italy), according to the CIE L*a*b* color System [37].

### 2.3. Questionnaire Structure

Each questionnaire (Table S2) consisted of three sections:

- Demographics: sex, age category, height, weight.
- Hedonic and emotional ratings: each participant provided ordinal ratings on a 0–10 scale for:
  - Hedonic judgments: wine alone (hedo), wine with music song 1 (hedo_mel), wine with music song 2 (hedo_upb).
  - Emotions: three positive emotions (pos_sur = positive surprise; pos1 = joy/happiness; pos2 = serenity/reliability) and three negative emotions (neg_sur = negative surprise; neg1 = disgust; neg2 = nostalgia/loss).
- Two open-ended text fields for positive and negative emotional comments.

Emotional items were presented as compound descriptors (e.g., ‘joy/happiness’, ‘serenity/reliability’) to enhance clarity for non-expert participants in a public setting. These measures are intended as descriptive affective labels rather than discrete psychological constructs.

### 2.4. Tasting Protocol and Experimental Control

At each event, four wines were served: three different wines plus a constant reference wine (hereafter “wineX”) that was served at all five events but in different positions within the tasting order (Table S3). The tasting order was intentionally arranged “in crescendo” (from lighter to more structured wines). Standard tasting portions (approximately 30 mL) were served. Participants were encouraged to rinse their mouths with water between wines. Ratings were collected within a short time window following each musical condition to minimize memory effects. Two music songs were used: song 1, melancholic/relaxing, and song 2, upbeat/motivational. The two musical excerpts were selected to induce contrasting affective states, corresponding, respectively, to a low-arousal/negative-valence (melancholic/relaxing) and a high-arousal/positive-valence (upbeat/motivational) emotional profile, in line with established frameworks in music-emotion research. All musical excerpts were performed live by a jazz trio and were purely instrumental, without lyrics, in order to minimize semantic priming effects and focus on emotional and affective modulation.

### 2.5. Statistical Analyses

Non-parametric and ordinal regression methods were used due to the ordinal nature of the data. Derived variables included improvement scores (impr_mel, impr_upb, impr_d). Tests included Kruskal–Wallis, Friedman, Wilcoxon with Holm correction, Spearman correlations with BH correction, and Cumulative Link Mixed Models (CLMM) for selected emotional variables. Reproducibility was ensured through R (ver. 4.5.2, R Foundation for Statistical Computing, Vienna, Austria) scripts and the statistical packages reported in detail in Table A1.

## 3. Results and Discussion

### 3.1. Statistical Model Design

From an overall perspective, our experimental design consists of a series of five events, each involving approximately 50 participants, with each participant completing a questionnaire yielding ordinal data. The statistical model most closely aligned with this structure is the Multi-Level (ML) model [38], which is typically applied to hierarchically organized data (e.g., multiple schools, each subdivided into classes, each class comprising several students). However, ML models usually assume that no individual belongs to more than one hierarchical unit; that is, the same student cannot be enrolled in multiple classes or schools.

In our case, by contrast, the same participant may attend more than one event; our dataset, therefore, presents a partially cross-classified structure, where tasters are not uniquely nested within a single event [39,40]. Given the ordinal nature of the hedonic responses, we applied a Cumulative Link Mixed Model (CLMM), which accommodates ordinal outcomes, allows for random effects and tolerates the existence of cross-classified entities. The CCLM includes:

- Random effects:
  ○ Participant ID (“id”).
  ○ Tasting event (“event”).
- Fixed effects:
  ○ Music condition: “cond”(“still” = no music, assumed as reference; “mel” = melancholic tune; “upb” = upbeat tune).
  ○ Tasting order within event: “tasord” (reference = position 1).
  ○ Wine identity: “wine” (reference = “wineX”).

Full results are presented in Figure S3; here we want to highlight the most important information.

The tasting conditions show that the music is strongly able to change the perceived hedonic evaluation of the wine: both melancholic and upbeat tunes result significantly different from the no-music condition with a probability less than 2 × 10^−16^; the effect is stronger (~0.97) for the upbeat tunes than for the melancholic ones (~0.72) (see the column “Estimate” of the Figure S3).

Most wines were rated significantly higher than wineX, with only a few showing no significant difference or getting a lower rate. As the wine set is limited (*n* = 16) and the study was not designed to associate wine characteristics with hedonic responses, we do not further interpret wine-level effects.

The effect of tasting order exhibits an unexpected non-monotonic pattern. Compared to the first position (i.e., the reference), all subsequent positions are associated with lower hedonic evaluations. In particular, positions 2 and 3 show a pronounced decrease in the estimated latent score (approximately −0.76 and −1.34, respectively), both highly significant (*p* = 3.6 × 10^−4^ and *p* = 1.18 × 10^−7^). In contrast, position 4 shows only a modest decrease (approximately −0.38), which does not reach conventional levels of statistical significance (*p* ≈ 0.06).

Regarding the random effects, the model estimates a substantial variance for the participant identifier (“id”; variance ≈ 1.35), indicating pronounced inter-individual variability in hedonic judgments across tasters. In contrast, the variance associated with the tasting event (‘event’) is estimated to be effectively zero, suggesting no detectable residual heterogeneity across events once participant- and wine-level effects are accounted for.

To further investigate the potential influence of tasters who participated in multiple events, we created a data subset excluding all raters involved in more than one session (approximately 5% of the total participants). We then re-estimated the Cumulative Link Mixed Model (CLMM) on this restricted dataset; full results are provided in Figure S4. The new model yields results that are broadly consistent with the original analysis:

- The music conditions show similar behavior and significance of the previous model, thus indicating that the cross-tasters do not influence the global evaluation of each wine
- The wines and the tasting order show a behaviour similar to that of the previous model, but the significance levels are lower
- The id-linked variance shows a very small difference (≈1.36 with respect to 1.35) thus indicating that multiple IDs reduce (as expected) the variability among tasters. The “event” variable continues showing zero variance, so the contribution of the event to the cumulative model is negligible.

It is also worth noting that the AIC (Akaike Information Criterion, a measure of the “goodness” of the model with respect to the data) value for this model (7524.28) is substantially lower than that of the original model (9170.76). However, since the reduced model is based on a smaller dataset, this difference in AIC should not be interpreted as evidence of improved fit by itself, but rather as a further suggestion that repeated participants do not destabilize the overall model structure.

To better assess the role of “tastord” (i.e., the tasting order in which wines were presented), we conducted a focused analysis using a subset of data that included only the reference wine, wineX. This wine was served in all five events, but at different positions within the tasting sequence: position 2 in Event 1, position 4 in Event 2, position 2 in Event 3, position 3 in Event 4, and position 1 in Event 5. By isolating wineX, we were able to examine the effect of tasting order, ruling out possible confounding contributions by different wine identities. The full results of this analysis are reported in Figure S5. Key findings include:

- The variance associated with the “id” random effect was markedly higher (4.36 compared to 1.36 in previous models), likely reflecting both the smaller dataset and increased variability among individual tasters when evaluating the same wine multiple times
- The variance for the “event” random effect remained near zero, providing further support for the negligible impact of event-level factors
- music conditions continued to elicit significantly higher hedonic ratings compared to the reference (“still” = no music) condition, with strong statistical significance (*p* ≈ 2 × 10^−10^)
- The effect of tasting order was negative overall. A significant decrease in rating was observed when wineX was served in position 3, suggesting a potential positional influence that will be discussed in the following using complementary analytical approaches.

Investigating tasting order effect is hard in our experimental design, since the tasting in all event were structured “in crescendo” (from lighter to more structured wines), where wines were presented in an order corresponding to their relative strength and complexity. The different positions of wineX across the events, depending on how its profile compared with the other wines presented that day. The order of presentation therefore could constitute a potential confounding variable: this can affect a sensitive model, like the CLMM discussed above.

Another approach to rule out both sources of spurious variation—event-related mood and presentation order—is a Kruskal–Wallis test [41,42] was conducted to compare the hedonic ratings of the reference wine (wineX) across the five events and across its different positions in the tasting sequence. It returned a non-significant result (Kruskal–Wallis χ^2^ = 6.31, df = 4, *p* = 0.177), indicating no evidence of systematic differences attributable to the event “mood” or to the serving order when wine identity is held constant. A simple linear regression (EQ 1) of hedonic score on presentation order for wineX estimated a negative slope (β_1_ ≈ −0.227), suggesting a small tendency for later presentation to be associated with lower scores, but this effect was not statistically significant (*p* = 0.108; R^2^ = 0.013).

(1) $$hedoval=\beta_{0}+\beta_{1}\;*\;tastord+\varepsilon$$

where *hedoval* is the hedonic score assigned to the wine; *tastord* is the serving order; $\beta_{0}$ is the intercept; $\beta_{1}$ the slope and ϵ the noise. This is consistent with the results of CLMMs applied to wineX data only: as the tasting order increases, the hedonic score seems to be penalized, even if this relationship is neither linear nor monotonic.

In this perspective, it might be interesting to evaluate the serving-order effect considering the full dataset (all wines across all events). In this case, the order of presentation strongly correlated with hedonic score (β_1_ = +0.428, *p* = 8.75 × 10^−12^). This finding reflects the deliberate “in crescendo” tasting design, where more structured wines were presented later and therefore perceived as more pleasing. The contrasting results for wineX versus the full dataset support the conclusion that (a) event-level contextual effects and serving order per se are not systematically biasing ratings for identical wines, and (b) the observed global association between order and hedonic score is mainly a by-product of the tasting sequencing of different wines. This result may appear to contradict the findings of the CLMM, which instead indicates a negative effect of tasting order. However, the discrepancy arises from the fundamentally different assumptions of the two approaches. The CLMM estimates the effect of tasting order while explicitly accounting for all fixed and random effects in the model, thereby isolating the contribution of tasting order itself. In contrast, the simple linear regression captures only the overall trend in the data, without controlling for potential confounding variables such as wine identity. The most plausible interpretation is that the observed increase in hedonic ratings along the tasting sequence—captured by the linear fit—is driven by the “in crescendo” design of the sessions. It is likely that these wines would have received similarly high ratings if they had been presented earlier in the sequence, suggesting that the positive trend reflects selection effects rather than a genuine effect of presentation order. Because hedonic ratings were collected sequentially (silence, first musical excerpt, second musical excerpt), potential confounding effects related to time, anchoring, or sensory adaptation cannot be entirely excluded. Accordingly, causal interpretations are avoided, and results are discussed in terms of modulation rather than direct causation.

### 3.2. Hedonic Differences Across Wines

A Kruskal–Wallis test performed on hedonic ratings across all wines rejected the null hypothesis of identical distributions (Kruskal–Wallis χ^2^ = 123.77, df = 19, *p* < 2.2 × 10^−16^), indicating significant differences in perceived pleasantness among the wines served. Given the limited characterization of wine attributes in the dataset, and the relatively small number of wines, extensive inference on wine-level determinants is unwarranted; we therefore have not further explored statistical comparisons among wines.

### 3.3. Music Effects on Hedonic Perception

The strong effect of music conditions, already highlighted by CLMM, is also confirmed by using the Friedman test [43]. As different wines were, in general, evaluated by different participants, it is not correct to perform a single Friedman test on all the results, but it is necessary to perform a single test for each wine. We then inferred the *p*-value of the whole dataset by applying the Stouffer method (weighted by the number of raters per wine) [44], obtaining a cumulative *p*-value of 6.19 × 10^−37^, demonstrating that listening to music significantly affects hedonic ratings at the series level [41,45].

The CLMM allows for a quantitative assessment of the effect of music on hedonic evaluations. Estimated marginal means (EMMs) were computed for each music condition, and pairwise contrasts were performed, yielding the results shown in Table 2.

Both the melancholic and upbeat music conditions significantly increased hedonic ratings compared to the still condition (*p* < 0.0001). Upbeat music was associated with higher ratings than melancholic music, with an estimated difference of approximately 0.25 on the latent scale (Figure S6).

The stronger and more consistent enhancement of hedonic ratings observed under upbeat/motivational music aligns with previous findings showing that music characterized by higher tempo and positive valence tends to amplify perceived pleasantness and sweetness of food and beverages. Fiegel et al. (2014) [29] demonstrated that background music genre significantly modulates overall flavor pleasantness, while Guedes et al. (2023) [30] reported that ‘sweet’ or uplifting music increases hedonic responses, particularly in products with moderate sensory intensity. In the wine domain, Billeci et al. (2025) [31] observed that live sonic seasoning can positively influence consumer experience in real tasting contexts, supporting the ecological validity of the present findings. Together, these studies suggest that upbeat/motivational music may act as an affective primer, biasing evaluative judgments toward higher liking through mood congruency mechanisms.

The present findings are consistent with wine-specific evidence showing that background music can systematically modulate hedonic wine evaluations. Early experimental studies demonstrated that wines are perceived as more pleasant when tasted in the presence of music whose emotional valence is congruent with the tasting context, suggesting that music operates as an affective crossmodal prime rather than as a purely decorative stimulus [46,47].

More recent work has further shown that music does not merely influence overall liking, but can bias the perception of wine-related sensory attributes such as sweetness, acidity, fruitiness, and persistence [48]. These effects appear to be mediated by emotional and semantic associations elicited by musical structure (tempo, mode, harmony), which interact with the cognitive appraisal of wine during tasting. In this framework, the stronger and more consistent hedonic enhancement observed under upbeat/motivational music in the present study can be interpreted as the result of mood-congruent facilitation of positive evaluative judgments, in line with crossmodal theories applied to wine perception.

We cannot construct an individual “musical preference profile” for each evaluator, as the number of observations per person is too limited. However, we can investigate whether distinct “preference personalities” exist regarding wine–music pairings by dividing the audience into four main categories:

- nomusic: when (impr_mel < 0) and (impr_upb < 0), meaning that the evaluator judged both musical excerpts as decreasing the wine’s hedonic rating.
- melanc: when (impr_mel > 0) and (impr_upb < 0), meaning that the evaluator judged mel to enhance the wine while upb diminished it.
- go: when (impr_mel < 0) and (impr_upb > 0), meaning that the evaluator judged mel to diminish the wine while upb improved it.
- allmusic: when (impr_mel > 0) and (impr_upb > 0), meaning that the music was judged to enhance the wine regardless of the emotional character of the excerpts.

The marked inter-individual variability observed in music-induced hedonic shifts is consistent with literature highlighting the role of personal sensitivity, expectations, and prior experience in crossmodal perception. Sinesio et al. (2021) [32] showed that contextual cues interact with individual traits to shape wine-evoked emotions and liking, while Motoki et al. (2023) [11] emphasized that crossmodal correspondences are probabilistic rather than universal. The predominance of the “allmusic” profile in the present study suggests that live music generally enhances enjoyment, yet the existence of minority profiles confirms that auditory modulation of taste is not homogeneous across consumers.

It turns out that approximately 70% of considered cases fell into the “allmusic” category (both songs improved hedonic ratings), while the remaining classes each accounted for roughly 10%. This predominance of “allmusic” responses argues for a broadly positive effect of live music on wine enjoyment that is not restricted to a small subset of wines or tasters. Moreover, these clusters about the effectiveness of music improvement do not show any association with evaluators’ personal characteristics (sex, age, etc.).

The substantial inter-individual variability observed in music-induced hedonic modulation agrees with previous wine-focused studies indicating that crossmodal effects persist even among experienced tasters. Wang and Spence (2017) [49] reported that professional wine assessors exhibited systematic changes in both hedonic and sensory ratings when wines were tasted with different soundtracks, indicating that musical modulation is not eliminated by analytical expertise.

These findings support the interpretation that variability in music–wine interactions reflects inherent differences in emotional sensitivity and crossmodal integration rather than a lack of tasting competence. Accordingly, the predominance of the allmusic profile observed in the present dataset suggests a generally facilitatory role of live music on wine enjoyment, while the existence of minority profiles confirms that auditory modulation of wine perception is probabilistic rather than universal.

Although the present design does not allow the isolation of specific musical properties (e.g., tempo versus mode), the more robust effect observed under upbeat/motivational music suggests that higher arousal and positive valence are the dominant drivers of music-induced hedonic enhancement.

### 3.4. Emotional Responses

**Figure 1:**
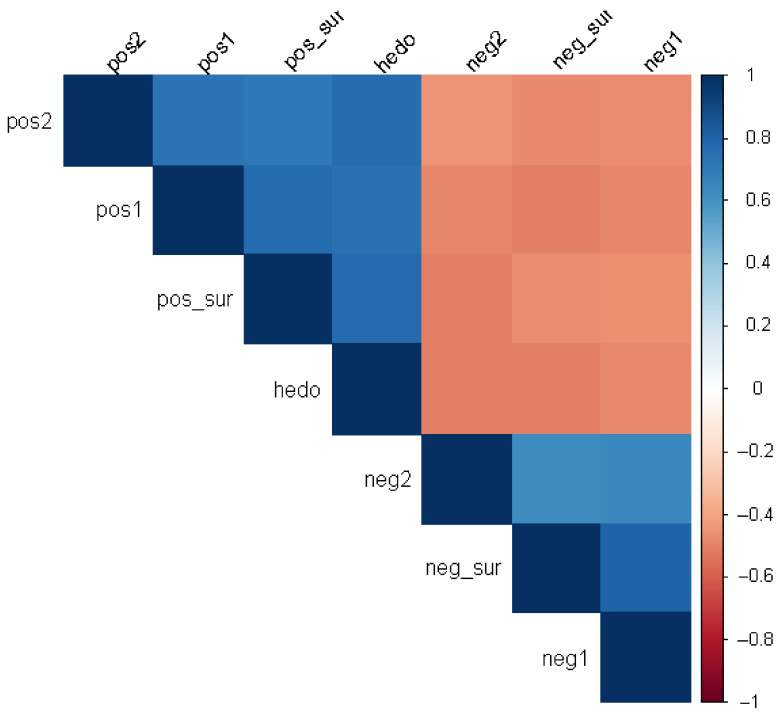
Heatmap of Spearman rho-coefficient for each pair of emotional variables.

**Figure 2:**
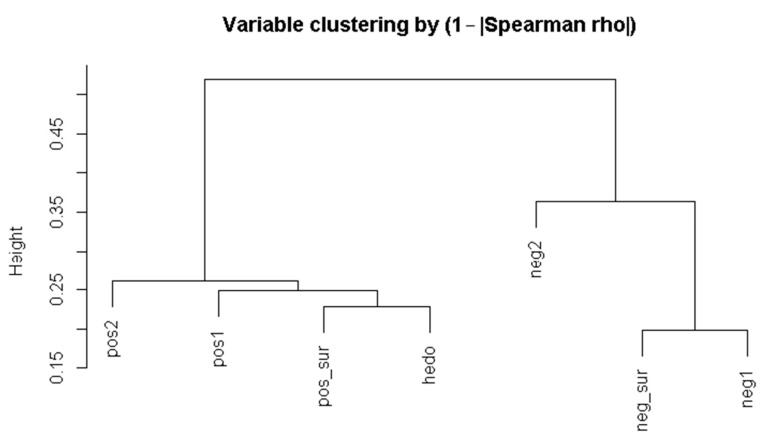
The dendrogram obtained by a hierarchical clustering of emotional variables, based on 1 − |rho-index|.

Emotion ratings reveal that positive emotions (pos_sur, pos1, pos2) have higher medians and more elevated quartiles compared to negative emotions, confirming that the set of wines was overall well received. Spearman correlations [50] show strong positive inter-correlations among positive emotions and strong associations among negative emotions; notably, positive surprise correlates strongly with hedonic rating (Spearman’s ρ ≈ 0.77), whereas positive and negative surprise are moderately anticorrelated (ρ ≈ −0.47) (Figure 1). Hierarchical clustering (Figure 2) based on correlations groups positive emotions together (with pos_sur closely related to hedonic score) and negative emotions together, with distinct patterns within each group.

The strong association between positive surprise and hedonic evaluation supports theoretical models in which surprise acts as a key driver of pleasure and memorability in sensory experiences. Beyond mood congruency, music may influence hedonic evaluation through attentional modulation, by increasing engagement with the tasting experience, semantic priming via culturally learned associations, and physiological arousal mechanisms that amplify reward processing. From a neurocognitive perspective, surprise has been linked to reward-related learning and heightened attentional engagement, particularly in multisensory contexts [19,23]. In food and beverage studies, emotional responses—especially those related to novelty and unexpected congruency—have been shown to predict liking beyond classical sensory descriptors [22,33]. Thus, the present findings reinforce the view that positive surprise represents a central affective mechanism through which music enhances wine appreciation. Within a predictive coding framework, positive surprise may reflect a reward prediction error, whereby unexpected congruency between auditory and gustatory inputs enhances pleasure and memorability.

The central role of positive surprise identified in the present study is strongly supported by wine sensory literature. Emotional responses related to novelty, expectation violation, and perceptual congruency have been shown to play a key role in shaping wine appreciation, often predicting liking more effectively than traditional sensory descriptors alone [33].

Dynamic sensory approaches further demonstrate that auditory context can influence the temporal evolution of wine perception. Studies using Temporal Dominance of Sensations (TDS) have shown that different musical environments modify the dominance patterns of sensory attributes during wine tasting, indicating that sound can alter not only the final judgment but also the unfolding perceptual experience [49,51,52].

Within this framework, positive surprise may represent a critical affective mechanism linking auditory stimulation and enhanced hedonic evaluation, by increasing attentional engagement and reward-related processing during multisensory wine tasting experiences.

Assessment of rater traits (gender, age, body_type, oeno_exp, music_exp) revealed generally weak correlations among traits (largest around 0.30, e.g., between age and experience variables), suggesting limited confounding. A Cumulative Link Mixed Model (CLMM) [53,54] applied to pos_sur with random intercepts for rater ID estimated (EQ 2) an intra-class correlation index (ICC) of approximately 12%, indicating meaningful between-rater variability.

(2) (ICC)=0.4310.431+π23 ≈0.116 

**Figure 3:**
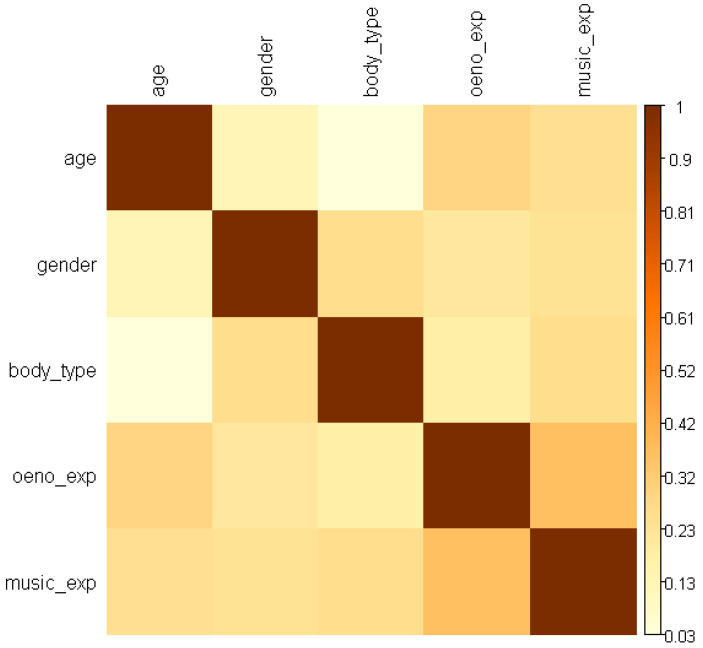
Heatmap of correlation values between rater traits.

Two modest associations emerged (Figure 3): (1) body_type showed a weak positive association with pos_sur (*p* ≈ 0.10 threshold), and (2) music experience exhibited a statistically detectable but subtle non-linear (quadratic) relationship with pos_sur, whereby intermediate levels of self-declared music experience (amateurs) tended to report slightly higher pos_sur than complete novices or professionals. Likelihood ratio testing suggested that inclusion of music_exp improved model fit marginally (*p* ≈ 0.08). Given the small numbers in extreme categories (e.g., professional musicians), these findings should be interpreted cautiously.

Overall, the data robustly indicate that live music modifies the hedonic appreciation of wine, with upbeat/motivational songs typically producing larger improvements than melancholic/relaxing songs. The observed effects are consistent across a variety of wines and tasters, although the magnitude of change is heterogeneous across individuals. The deliberate tasting order (“in crescendo”) creates a strong confound between wine identity and presentation order in the full dataset, while apparently there is no effect of the “event” variable. Emotional data suggest that positive surprise is a central component of hedonic appreciation for these tastings. Accordingly, emotional findings should be interpreted as reflecting self-reported affective impressions rather than validated psychometric dimensions.

Future work would benefit from a larger sample of repeat raters, richer wine metadata (e.g., wine style, alcohol, body), and Bayesian models that explicitly model rater- and wine-level heterogeneity, in particular, by explicating the contribution given by repeated tasters on the model. Nevertheless, the limited number of repeat observations per evaluator reduces statistical power for detecting moderator effects, and the absence of strong associations with personal characteristics should be interpreted with caution.

## 4. Conclusions

This study demonstrates that live music systematically influences the wine perception, affecting both hedonic evaluations and emotional responses, in real-world tasting contexts. Across five public tasting events, participants consistently rated wines as more enjoyable when accompanied by music than when tasted in silence. A clear asymmetry emerged between musical conditions. Upbeat/motivational music produced stronger and more consistent hedonic improvements than melancholic/relaxing music, both in terms of frequency of statistically significant effects and mean improvement scores. Although the experimental design does not allow for the isolation of individual musical parameters (e.g., tempo, mode, or harmonic structure), this pattern suggests that higher arousal and positive emotional valence are dominant drivers of music-induced hedonic enhancement. Emotional analysis indicated that positive surprise emerged as a central mediator of increased liking, highlighting the role of affective mechanisms in shaping multisensory experiences. These findings align with evolutionary and neuroscientific perspectives, suggesting that olfactory and gustatory perception are deeply intertwined with emotional processing, and that crossmodal integration can meaningfully alter subjective experience [12,13,30,55,56,57,58]. Although wines of different styles were included, the present design was not intended to formally test interactions between wine style (e.g., red vs. white, structure, tannin level) and musical condition. Future studies specifically designed to examine how wine structure, aromatic intensity, or style interact with musical parameters would further refine our understanding of auditory–gustatory congruency.

Furthermore, the results underscore the importance of ecological validity in sensory research: by examining consumer responses in real-world tasting contexts, this study captures the complexity of wine appreciation as a multisensory, cognitive, and emotional phenomenon. From an applied perspective, the findings suggest that strategic use of music can enhance consumer enjoyment and engagement in hospitality and wine marketing contexts. By situating these results within the broader framework of crossmodal and affective sensory science, the study supports the notion that auditory cues can systematically bias evaluative processes through emotional mediation rather than through direct alteration of sensory attributes. This interpretation is consistent with the literature on multisensory food perception [48,55,56] and suggests that music functions as a contextual enhancer that shapes meaning, expectation, and affect during wine tasting.

From an applied perspective, these findings suggest that upbeat, high-arousal music may be strategically employed during tastings of approachable or entry-level wines to enhance enjoyment, while more restrained musical settings may be preferable for structured or contemplative tasting formats.

Overall, these findings reinforce the notion that wine appreciation is not solely determined by chemical composition but emerges from an integrated perceptual and emotional experience. Live music appears to function as a contextual enhancer that shapes hedonic evaluation primarily through emotional mediation rather than through direct sensory alteration.

## Figures and Tables

**Table 1:** Code and the information about the wines used.

Wine Code	Type	Name	Cellar/Producer	Vintage	Variety	Denomination/Region
WineX	Red	Tavernello rosso	Cantine Caviro	2024	not specified	Table wine (Emilia-Romagna)
WineA	Rosé	L’Altro Punto di Vista	Podere La Chiesa	2023	Sangiovese (100%)	DOC-Terre di Pisa
WineB	Red	Terre di Casanova	Podere La Chiesa	2023	Sangiovese (100%)	Chianti DOCG
WineC	Red	Sabiniano di Casanova	Podere La Chiesa	2021	Sangiovese (60%), Cabernet Sauvignon (25%), Merlot (15%)	DOC-Terre di Pisa
WineD	White	Pinot Grigio “Borgo Tesis”	Fantinel	2023	Pinot Gris (100%)	DOC-Friuli Grave
WineE	Sparkling	Talento Brut Metodo Classico Etichetta Argento	Pittaro	2022	Chardonnay (80%) e Pinot Blanc (20%)	DOC-Friuli Grave
WineF	White	Friulano	Livio Felluga	2023	Friulano (100%)	DOC-Friuli Colli Orientali
WineG	White	Sauvignon	Pitars	2023	Sauvignon Blanc (100%)	DOC-Friuli Grave
WineH	Red	Schioppettino	La Sclusa	2022	Schioppettino (100%)	DOC-Friuli Colli Orientali
WineI	Red	Refosco dal Peduncolo Rosso	Ca’ Bolani	2022	Refosco dal peduncolo rosso (100%)	DOC-Friuli Aquileia
WineL	White	Villa Antinori	Marchesi Antinori	2024	Trebbiano Toscano, Malvasia, Pinot Blanc, Pinot Gris, Riesling (% not specified)	IGT-Toscana
WineM	Rosé	Scalabrone	Tenuta Guado al Tasso—Marchesi Antinori	2024	Cabernet sauvignon (40%), Merlot (30%), Syrah (30%)	DOC-Bolgheri
WineN	Red	Achelo	Tenuta La Braccesca—Marchesi Antinori	2023	Syrah (100%)	DOC-Cortona
WineO	Red	Il Grullaio	Usiglian del Vescovo	2023	Cabernet Sauvignon (50%), Merlot (50%)	IGT-Costa Toscana
WineP	Red	Valle delle Stelle	Brancatelli	2021	100% Cabernet Sauvignon	IGT-Toscana
WineQ	Red	Midnight Star	Usiglian del Vescovo	2018	Sangiovese (100%)	IGT-Toscana

**Table 2:** Estimated pairwise contrasts for music conditions.

Contrast	Estimate	SE	df	z-Ratio	*p*-Value
still–mel	−0.7192256	0.08365979	Inf	−8.597	<0.0001
still–upb	−0.9731513	0.08532845	Inf	−11.405	<0.0001
mel–upb	−0.2539257	0.08809851	Inf	−2.882	0.0110

## Data Availability

The original contributions presented in this study are included in the article/Supplementary Material. Further inquiries can be directed to the corresponding author.

## Supplementary Materials

The following supporting information can be downloaded at: https://www.mdpi.com/article/10.3390/foods15030504/s1, Figure S1: Photo of public tasting event entitled “*5 Wednesdays of Emotions*”; Figure S2: PERMANOVA results utilized for longitudinal effect assessment; Figure S3: CCLM applied to full dataset; Figure S4: CCLM applied to restricted dataset (excluding all raters involved in more than one session); Figure S5: CLMM applied to Wine X; Figure S6: Estimated marginal means of evaluation condition; Table S1: Color and Chemical parameters of wine; Table S2: Questionnaire questions and the associated variables used in R scripts; Table S3: Tasting order of the different wine during the 5 events.

## Appendix A

**Table A1:** R package and version used in the study with references.

**R Package**	**Version**	**Reference**
corrplot	0.95	[59]
gridExtra	2.3	[60]
MASS	7.3-65	[61]
metap	1.13	[62]
ordinal	12-4.1	[63]
pairwiseAdonis	0.4	[64]
polycor	0.8-1	[65]
psych	2.5.6	[66]
reshape2	1.4.5	[67]
tidyverse	2.0.0	[68]
vegan	2.7-2	[69]

## References

[1] Parr W.V., Mouret M., Blackmore S., Pelquest-Hunt T., Urdapilleta I. (2011). Representation of complexity in wine: Influence of expertise. Food Qual. Prefer..

[2] Taglieri I., Sanmartin C., Bianchi A., Dìaz-Guerrero P., Ferroni G., Tonacci A., Venturi F. (2025). Emotional impact of red wine assessed through a multidisciplinary approach. Appl. Food Res..

[3] Bianchi A., Taglieri I., Macaluso M., Sanmartin C., Zinnai A., Venturi F. (2023). Effect of Different Packaging Strategies on the Secondary Shelf Life of Young and Structured Red Wine. Foods.

[4] Bianchi A., Pettinelli S., Pittari E., Paoli L., Sanmartin C., Pons A., Mencarelli F., Piombino P. (2025). Accelerated oxygenation for the production of fortified (mystelle-type) sweet wines: Effects on the chemical and flavor profile. J. Sci. Food Agric..

[5] Auffarth B. (2013). Understanding smell—The olfactory stimulus problem. Neurosci. Biobehav. Rev..

[6] Ferreira V., Reynolds A.G. (2010). 1—Volatile aroma compounds and wine sensory attributes. Managing Wine Quality.

[7] Sáenz-Navajas M.-P., Campo E., Culleré L., Fernández-Zurbano P., Valentin D., Ferreira V. (2010). Effects of the Nonvolatile Matrix on the Aroma Perception of Wine. J. Agric. Food Chem..

[8] Ferreira V., de la Fuente A., Sáenz-Navajas M.P., Reynolds A.G. (2022). Wine aroma vectors and sensory attributes. Managing Wine Quality.

[9] Yuan W., Guo F., Li M., Song H. (2024). Effects of sensory cues on consumers’ wine taste perceptions and behavior: Evidence from a wine-tasting experiment. Int. J. Contemp. Hosp. Manag..

[10] Bianchi A., Pacifico S., Santini G., Pettinelli S., Alfieri G., Modesti M., Bellincontro A., Sanmartin C., Pittari E., Piccolella S. (2025). Carbonic or nitrogen maceration of wine grape: Biochemical differences of grape and wine using destructive and non-destructive approach. Food Chem..

[11] Motoki K., Marks L.E., Velasco C. (2023). Reflections on Cross-Modal Correspondences: Current Understanding and Issues for Future Research. Multisens. Res..

[12] Lalanne C., Lorenceau J. (2004). Crossmodal integration for perception and action. J. Physiol..

[13] Jessen S., Kotz S.A. (2013). On the role of crossmodal prediction in audiovisual emotion perception. Front. Hum. Neurosci..

[14] Di Stefano N., Spence C. (2025). Perceiving temporal structure within and between the senses: A multisensory/crossmodal perspective. Atten. Percept. Psychophys..

[15] Tonacci A., Billeci L., Burrai E., Sansone F., Conte R. (2019). Comparative Evaluation of the Autonomic Response to Cognitive and Sensory Stimulations through Wearable Sensors. Sensors.

[16] White T.L., Thomas-Danguin T., Olofsson J.K., Zucco G.M., Prescott J. (2020). Thought for food: Cognitive influences on chemosensory perceptions and preferences. Food Qual. Prefer..

[17] Izard C.E. (2009). Emotion theory and research: Highlights, unanswered questions, and emerging issues. Annu. Rev. Psychol..

[18] Tonacci A., Billeci L., Di Mambro I., Marangoni R., Sanmartin C., Venturi F., Di Mambro I., Marangoni R., Sanmartin C., Venturi F. (2021). Wearable sensors for assessing the role of olfactory training on the autonomic response to olfactory stimulation. Sensors.

[19] Kryklywy J.H., Ehlers M.R., Anderson A.K., Todd R.M. (2020). From Architecture to Evolution: Multisensory Evidence of Decentralized Emotion. Trends Cogn. Sci..

[20] Joy A., Charters S., Wang J.J., Grohmann B. (2020). A multi-sensory and embodied understanding of wine consumption. J. Wine Res..

[21] Malfeito-Ferreira M. (2023). Fine wine recognition and appreciation: It is time to change the paradigm of wine tasting. Food Res. Int..

[22] Rodrigues Á. (2025). Tourists’ Sensory Engagement and Emotional Response in Blind Wine Tasting. Gastron. Tour..

[23] Wolff M., Morceau S., Folkard R., Martin-Cortecero J., Groh A. (2021). A thalamic bridge from sensory perception to cognition. Neurosci. Biobehav. Rev..

[24] Soudry Y., Lemogne C., Malinvaud D., Consoli S.-M., Bonfils P. (2011). Olfactory system and emotion: Common substrates. Eur. Ann. Otorhinolaryngol. Head Neck Dis..

[25] De Luca R., Botelho D. (2021). The unconscious perception of smells as a driver of consumer responses: A framework integrating the emotion-cognition approach to scent marketing. AMS Rev..

[26] Álvarez-Pato V.M., Sánchez C.N., Domínguez-Soberanes J., Méndoza-Pérez D.E., Velázquez R. (2020). A Multisensor Data Fusion Approach for Predicting Consumer Acceptance of Food Products. Foods.

[27] Morquecho-Campos P., de Graaf K., Boesveldt S. (2020). Smelling our appetite? The influence of food odors on congruent appetite, food preferences and intake. Food Qual. Prefer..

[28] Baccarani A., Brand G., Dacremont C., Valentin D., Brochard R. (2021). The influence of stimulus concentration and odor intensity on relaxing and stimulating perceived properties of odors. Food Qual. Prefer..

[29] Fiegel A., Meullenet J.-F., Harrington R.J., Humble R., Seo H.-S. (2014). Background music genre can modulate flavor pleasantness and overall impression of food stimuli. Appetite.

[30] Guedes D., Prada M., Lamy E., Garrido M.V. (2023). Sweet music influences sensory and hedonic perception of food products with varying sugar levels. Food Qual. Prefer..

[31] Billeci L., Sanmartin C., Tonacci A., Taglieri I., Ferroni G., Marangoni R., Venturi F. (2025). Wearable sensors to measure the influence of sonic seasoning on wine consumers in a live context: A preliminary proof-of-concept study. J. Sci. Food Agric..

[32] Sinesio F., Moneta E., Di Marzo S., Zoboli G.P., Abbà S. (2021). Influence of wine traits and context on liking, intention to consume, wine-evoked emotions and perceived sensory sensations. Food Qual. Prefer..

[33] Galmarini M.V., Silva Paz R.J., Enciso Choquehuanca D., Zamora M.C., Mesz B. (2021). Impact of music on the dynamic perception of coffee and evoked emotions evaluated by temporal dominance of sensations (TDS) and emotions (TDE). Food Res. Int..

[34] Venturi F., Tonacci A., Ascrizzi R., Sansone F., Conte R., Pala A.P., Tarabella A., Sanmartin C., Taglieri I., Marangoni R. (2024). “CANTINA 5.0”—A Novel, Industry 5.0-Based Paradigm Applied to the Winemaking Industry in Italy. Appl. Sci..

[35] Santini G., Bianchi A., Pettinelli S., Modesti M., Cerreta R., Bellincontro A. (2023). Air speed and plastic crate vent-holes for wine grape quality during postharvest dehydration: Commercial and laboratory studies. J. Sci. Food Agric..

[36] Bianchi A., Taglieri I., Rimbotti Antinori V., Palla F., Macaluso M., Ferroni G., Sanmartin C., Venturi F., Zinnai A. (2021). A statistical approach to describe the ripening evolution of sangiovese grapes coming from different chianti classico sub-areas. Foods.

[37] Bianchi A., Taglieri I., Venturi F., Sanmartin C., Ferroni G., Macaluso M., Palla F., Flamini G., Zinnai A. (2022). Technological Improvements on FML in the Chianti Classico Wine Production: Co-Inoculation or Sequential Inoculation?. Foods.

[38] Raudenbush S.W., Bryk A.S., Raudenbush S.W., Bryk A.S. (2002). Hierarchical Linear Models: Applications and Data Analysis Methods.

[39] Dedrick R.F., Ferron J.M., Hess M.R., Hogarty K.Y., Kromrey J.D., Lang T.R., Niles J.D., Lee R.S. (2009). Multilevel Modeling: A Review of Methodological Issues and Applications. Rev. Educ. Res..

[40] Fernández-Castilla B., Jamshidi L., Declercq L., Beretvas S.N., Onghena P., Van den Noortgate W. (2020). The application of meta-analytic (multi-level) models with multiple random effects: A systematic review. Behav. Res. Methods.

[41] Hollander M., Wolfe D.A., Chicken E. (2013). Nonparametric Statistical Methods.

[42] Kruskal W.H., Wallis W.A. (1952). Use of Ranks in One-Criterion Variance Analysis. J. Am. Stat. Assoc..

[43] Friedman M. (1937). The Use of Ranks to Avoid the Assumption of Normality Implicit in the Analysis of Variance. J. Am. Stat. Assoc..

[44] Hedges L.V., Olkin I. (2014). Statistical Methods for Meta-Analysis.

[45] Lehmann E.L., Romano J.P. (2005). Testing Statistical Hypotheses.

[46] Pearce M.T. (2023). Music Perception. Oxf. Res. Encycl. Psychol..

[47] North A.C., Hargreaves D.J., McKendrick J. (1999). The influence of in-store music on wine selections. J. Appl. Psychol..

[48] Spence C., Wang Q. (2015). Wine and music (II): Can you taste the music? Modulating the experience of wine through music and sound. Flavour.

[49] Wang Q., Spence C. (2017). Assessing the influence of music on wine perception among wine professionals. Food Sci. Nutr..

[50] Spearman C., Jenkins J.J., Paterson D.G. (1961). The Proof and Measurement of Association Between Two Things. Studies in Individual Differences: The Search for Intelligence.

[51] Galmarini M.V., Loiseau A.-L., Debreyer D., Visalli M., Schlich P. (2017). Use of Multi-Intake Temporal Dominance of Sensations (TDS) to Evaluate the Influence of Wine on Cheese Perception. J. Food Sci..

[52] Mesz B., Trevisan M.A., Sigman M. (2011). The Taste of Music. Perception.

[53] Agresti A., Kateri M., Lovric M. (2025). Categorical Data Analysis. International Encyclopedia of Statistical Science.

[54] Grilli L., Rampichini C., Kenett R.S., Salini S. (2011). Multilevel models for ordinal data. Modern Analysis of Customer Surveys: With Applications Using R.

[55] Verhagen J.V., Engelen L. (2006). The neurocognitive bases of human multimodal food perception: Sensory integration. Neurosci. Biobehav. Rev..

[56] Seo H.-S., Hummel T., Buettner A. (2017). Cross-Modal Integration in Olfactory Perception. Springer Handbook of Odor.

[57] Zhang R., Jia W., Shu J., Zou L., Shi L. (2024). Flavor Experiences Augmentation Strategy for Fermented Dairy Products: Perspective of Multimodal Perception, Starter and Enhancer, and Processing. Food Rev. Int..

[58] Dikecligil G.N., Gottfried J.A., Skov M., Nadal M. (2022). Odour aesthetics: Hedonic perception of olfactory stimuli. The Routledge International Handbook of Neuroaesthetics.

[59] Wei T., Simko V.R. (2024). R Package, “Corrplot”: Visualization of a Correlation Matrix.

[60] Auguie B., Antonov A. (2017). R Package, Package ‘gridExtra’: Miscellaneous Functions for” Grid” Graphics.

[61] Venables W.N., Ripley B.D. (2002). Modern Applied Statistics with S.

[62] Dewey M. (2025). R Package, metap: Meta-Analysis of Significance Values.

[63] Christensen R.H.B. (2023). R Package, Package “ordinal”: Regression Models for Ordinal Data.

[64] Martinez Arbizu P. (2020). R Package, pairwiseAdonis: Pairwise Multilevel Comparison Using Adonis.

[65] Fox J. (2022). R Package, polycor: Polychoric and Polyserial Correlations.

[66] Revelle W. (2020). R Package, psych: Procedures for Psychological, Psychometric, and Personality Research.

[67] Wickham H. (2007). Reshaping data with the reshape package. J. Stat. Softw..

[68] Wickham H., Averick M., Bryan J., Chang W., McGowan L.D., François R., Grolemund G., Hayes A., Henry L., Hester J. (2019). Welcome to the Tidyverse. J. Open Source Softw..

[69] Oksanen J., Simpson G.L., Blanchet F.G., Kindt R., Legendre P., Minchin P.R., O’Hara R.B., Solymos P., Stevens M.H.H., Szoecs E. (2001). R Package, Vegan: Community Ecology Package.

