# Supplementary material for: Influence of Live Music and Tasting Assessment on Hedonic and Emotional Responses of Wine in Public Tasting Events
Source: Foods. 2026 Feb 1;15(3):504. doi: 10.3390/foods15030504 (PMC12896400; doi:10.3390/foods15030504)
Supplement: Supplementary file 1 [file foods-15-00504-s001.zip › foods-4088973-supplementary.pdf]

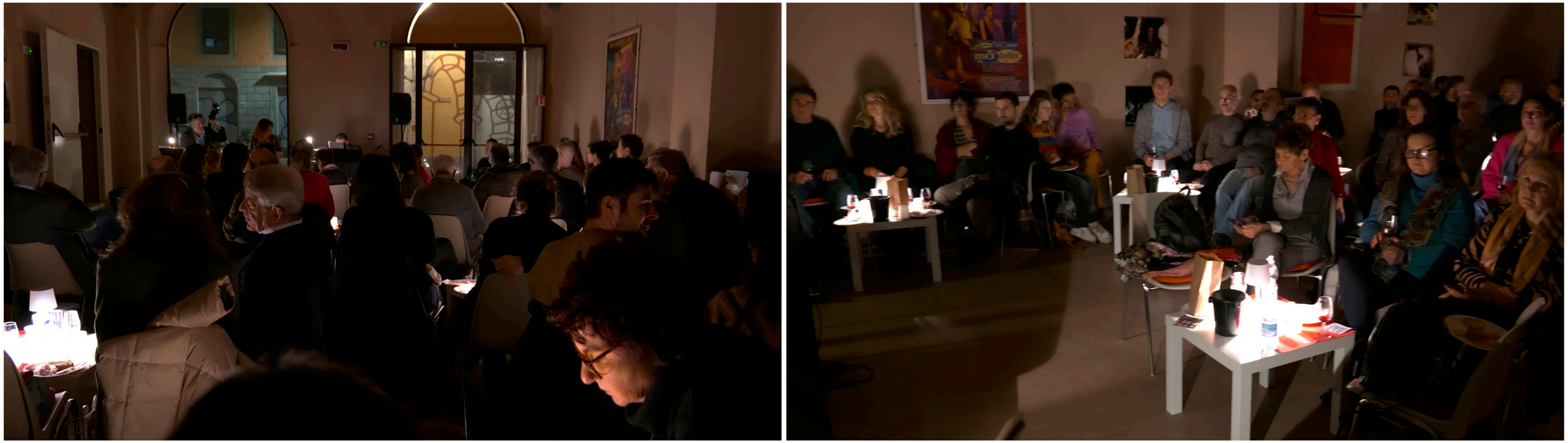

Figure S1. Photo of public tasting event entitled "*5 Wednesdays of Emotions*".

```
Permutation test for adonis under reduced model
Permutation: free
Number of permutations: 999

adonis2(formula = hedoX_carnall$hedo ~ hedoX_carnall$event, method = "gower")
         Df SumOfSqs      R2      F Pr(>F)
Model     3  0.09286 0.04693 0.2626  0.832
Residual 16  1.88571 0.95307
Total    19  1.97857 1.00000
```

Figure S2. PERMANOVA results utilized for longitudinal effect assessment.

Cumulative Link Mixed Model fitted with the Laplace approximation

formula: hedo ~ cond + wine + tasord + (1 | event) + (1 | id)
data: hedolong

link	threshold	nobs	logLik	AIC	niter	max.grad	cond.H
logit	flexible	2388	-4553.38	9170.76	624(47464)	2.04e-03	6.4e+02

Random effects:

Groups	Name	Variance	Std.Dev.
id	(Intercept)	1.351	1.162
event	(Intercept)	0.000	0.000

Number of groups: id 173, event 5

Coefficients:

		Estimate	Std. Error	z value	Pr(>|z|)
cond	mel	0.71923	0.08366	8.597	< 2e-16 ***
cond	upb	0.97315	0.08533	11.405	< 2e-16 ***
wine	wineA	-1.19591	0.25086	-4.767	1.87e-06 ***
wine	wineB	1.51652	0.28223	5.373	7.73e-08 ***
wine	wineC	1.27806	0.25533	5.005	5.57e-07 ***
wine	wined	-0.20799	0.17969	-1.157	0.247082
wine	wine	0.42732	0.24527	1.742	0.081460 .
wine	wineF	2.01268	0.27819	7.235	4.65e-13 ***
wine	wineG	-0.04936	0.25519	-0.193	0.846644
wine	wineH	1.50560	0.28606	5.263	1.42e-07 ***
wine	wineI	1.25060	0.25244	4.954	7.27e-07 ***
wine	wineL	-1.31924	0.24636	-5.355	8.56e-08 ***
wine	wineM	-0.51453	0.23942	-2.149	0.031632 *
wine	wineN	0.98763	0.24654	4.006	6.17e-05 ***
wine	wineO	1.03515	0.25773	4.016	5.91e-05 ***
wine	wineP	2.22434	0.28882	7.701	1.35e-14 ***
wine	wineQ	1.65849	0.25939	6.394	1.62e-10 ***
tasord	2	-0.75807	0.21256	-3.566	0.000362 ***
tasord	3	-1.33779	0.25257	-5.297	1.18e-07 ***
tasord	4	-0.37793	0.20101	-1.880	0.060083 .

Signif. codes: 0 ‘***’ 0.001 ‘**’ 0.01 ‘*’ 0.05 ‘.’ 0.1 ‘ ’ 1

Figure S3.CCLM applied to full dataset.

Cumulative Link Mixed Model fitted with the Laplace approximation

formula: hedo ~ cond + wine + tasord + (1 | event) + (1 | id)

data: hedolong_singleid

link	threshold	nobs	logLik	AIC	niter	max.grad	cond.H
logit	flexible	1944	-3730.14	7524.28	6174(36238)	7.62e-04	1.7e+03

Random effects:

Groups	Name	Variance	Std.Dev.
id	(Intercept)	1.357	1.165
event	(Intercept)	0.000	0.000

Number of groups: id 162, event 5

Coefficients:

		Estimate	Std. Error	z value	Pr(>|z|)
cond	mel	0.73666	0.09961	7.395	1.41e-13 ***
cond	upb	0.96463	0.10032	9.616	< 2e-16 ***
wine	wineA	-1.39780	0.39476	-3.541	0.000399 ***
wine	wineB	1.14066	0.37738	3.023	0.002506 **
wine	wineC	1.26415	0.38035	3.324	0.000888 ***
wine	wined	-0.54536	0.39715	-1.373	0.169701
wine	wine	0.12775	0.33202	0.385	0.700396
wine	wineF	1.54963	0.38181	4.059	4.94e-05 ***
wine	wineG	-0.08555	0.37497	-0.228	0.819530
wine	wineH	1.31808	0.35952	3.666	0.000246 ***
wine	wineI	1.27326	0.36119	3.525	0.000423 ***
wine	wineL	-1.56288	0.39186	-3.988	6.65e-05 ***
wine	wineM	-0.37402	0.32335	-1.157	0.247386
wine	wineN	1.04297	0.37781	2.761	0.005770 **
wine	wineO	0.83867	0.34379	2.439	0.014710 *
wine	wineP	2.13399	0.39216	5.442	5.28e-08 ***
wine	wineQ	1.82103	0.39563	4.603	4.17e-06 ***
tasord	2	-0.85858	0.34716	-2.473	0.013393 *
tasord	3	-1.27604	0.39295	-3.247	0.001165 **
tasord	4	-0.59260	0.39393	-1.504	0.132497

Signif. codes: 0 ‘***’ 0.001 ‘**’ 0.01 ‘*’ 0.05 ‘.’ 0.1 ‘ ’ 1

Figure S4.CCLM applied to restricted dataset (excluding all raters involved in more than one session).

```
Cumulative Link Mixed Model fitted with the Laplace approximation

formula: hedo ~ cond + tasord + (1 | event) + (1 | id)
data:    hedo_winex
```

link	threshold	nobs	logLik	AIC	niter	max.grad	cond.H
logit	flexible	597	-1139.14	2312.28	2383(14942)	3.07e-04	3.2e+02

Random effects:

Groups	Name	Variance	Std.Dev.
id	(Intercept)	4.365	2.089
event	(Intercept)	0.000	0.000

Number of groups: id 173, event 5

Coefficients:

		Estimate	Std. Error	z value	Pr(>|z|)
cond	mel	1.1642	0.1865	6.241	4.35e-10 ***
cond	upb	1.3429	0.1914	7.016	2.28e-12 ***
tasord	2	-0.9088	0.3802	-2.391	0.0168 *
tasord	3	-1.6295	0.4135	-3.940	8.14e-05 ***
tasord	4	-0.3788	0.4183	-0.906	0.3651

```
---
Signif. codes:  0 ‘***’ 0.001 ‘**’ 0.01 ‘*’ 0.05 ‘.’ 0.1 ‘ ’ 1
```

Figure S5. CLMM applied to Wine X.

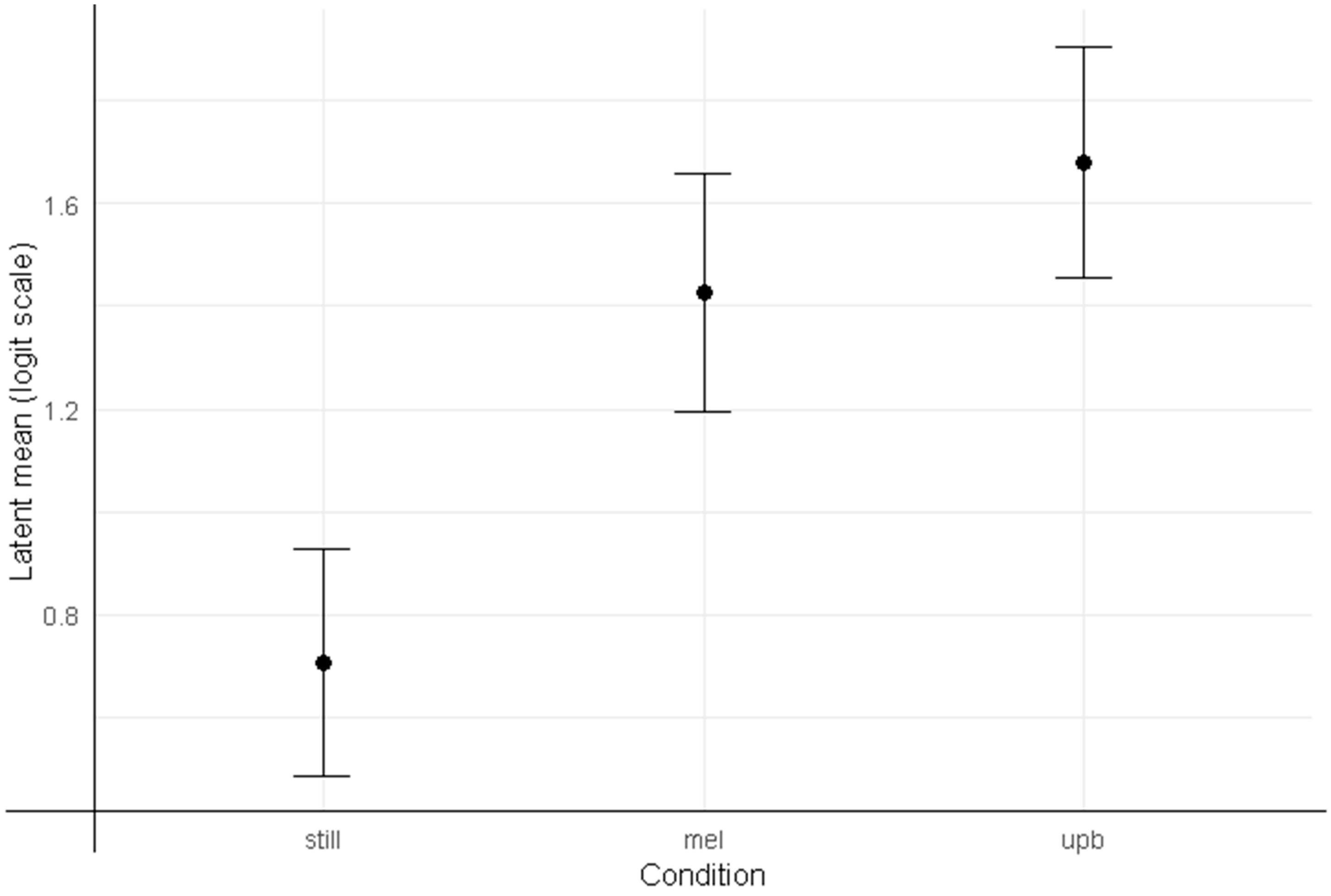

Figure S6. Estimated marginal means of evaluation condition.

Table S1. Color and Chemical parameters of wine.

Wine code	Alcohol (% V/V)	Sugars (g hexoses/L)	pH	Titratable acidity (g tartaric acid/L)	Volatile acidity (g acetic acid/L)	Malic acid (g/L)	Latic acid (g/L)	Total extract (g/L)	Glycerol (g/L)	Total Anthocyanins (mg malvidin/L)	Total polyphenols (mg gallic acid /L)	L*	a*	b*
WineX	11.28±0.07	6.95±0.08	3.49±0.04	5.10±0.04	0.49±0.02	0.03±0.01	1.03±0.03	34.01±0.15	7.29±0.03	301.50±5.70	2283.50±21.92	4.34±0.01	30.65±0.10	12.07±0.19
WineA	13.17±0.07	2.13±0.05	3.28±0.03	6.71±0.07	0.46±0.01	1.32±0.03	0.21±0.01	27.75±0.05	7.18±0.04	195.10±18.20	1118.00±1.41	45.49±0.05	11.61±0.07	23.09±0.05
WineB	14.19±0.02	1.42±0.10	3.66±0.02	5.74±0.08	0.92±0.05	n.d.	1.49±0.02	33.13±0.30	9.50±0.06	413.00±2.82	3142.00±48.08	1.59±0.07	31.83±0.16	8.29±0.08
WineC	14.17±0.07	0.99±0.04	3.67±0.07	5.63±0.04	0.94±0.02	n.d.	1.44±0.02	32.89±0.09	9.49±0.04	467.50±3.53	3642.50±23.33	0.02±0.01	27.87±0.08	3.84±0.03
WineD	12.58±0.02	1.13±0.13	3.31±0.01	5.37±0.02	0.29±0.01	1.87±0.02	n.d.	21.22±0.11	5.82±0.02	n.d.	736.3±45.35	59.18±0.06	−1.49±0.06	3.33±0.03
WineE	12.25±0.04	7.14±0.14	3.13±0.02	6.36±0.08	0.46±0.03	n.d.	2.34±0.07	28.10±0.17	5.82±0.04	n.d.	734.00±28.28	58.77±0.07	−3.02±0.06	6.49±0.15
WineF	12.93±0.07	3.05±0.07	3.37±0.01	5.93±0.07	0.51±0.02	n.d.	1.56±0.07	29.93±0.09	8.63±0.04	n.d.	784.50±4.95	58.24±0.02	−3.32±0.02	7.52±0.03
WineG	12.70±0.06	2.99±0.10	3.42±0.02	6.46±0.06	0.36±0.03	2.24±0.07	0.30±0.02	29.68±0.06	7.14±0.02	n.d.	823.66±9.50	56.31±0.05	−2.23±0.09	6.57±0.12
WineH	13.43±0.06	2.45±0.04	3.48±0.01	5.23±0.06	0.54±0.04	0.05±0.01	1.04±0.01	27.10±0.18	7.39±0.08	368.67±3.51	1983.66±2.88	2.35±0.09	45.38±0.08	17.99±0.07
WineI	12.93±0.07	2.41±0.13	3.59±0.01	5.47±0.05	0.60±0.01	n.d.	1.95±0.03	30.93±0.14	8.42±0.04	466.00±6.24	2337.33±29.02	1.55±0.06	41.56±0.13	9.13±0.25
WineL	12.03±0.07	2.49±0.19	3.24±0.04	5.20±0.03	0.28±0.01	1.38±0.02	0.11±0.02	21.13±0.18	6.04±0.06	n.d.	795.00±38.18	59.06±0.03	-2.21±0.06	3.70±0.02
WineM	12.37±0.04	0.47±0.07	3.24±0.01	5.44±0.08	0.37±0.02	1.26±0.02	0.13±0.02	19.62±0.09	6.22±0.07	148.70±0.00	815.50±7.77	55.04±0.05	5.55±0.06	7.91±0.07
WineN	14.06±0.07	0.99±0.01	3.82±0.01	4.70±0.02	0.69±0.03	n.d.	1.46±0.01	32.88±0.06	9.76±0.04	419.00±3.41	2398.50±6.36	−0.33±0.02	26.01±0.03	1.95±0.06
WineO	13.74±0.10	0.44±0.03	3.52±0.01	6.68±0.07	0.75±0.02	0.48±0.01	1.14±0.04	32.63±0.09	11.44±0.05	415.50±7.77	2808.50±10.60	−2.61±0.06	23.16±0.05	−1.10±0.02
WineP	13.91±0.07	0.71±0.01	3.65±0.01	5.38±0.04	0.66±0.04	n.d.	1.23±0.01	34.11±0.16	10.12±0.02	408.50±6.36	3256.504.90	−1.31±0.06	27.17±0.03	2.32±0.03
WineQ	13.94±0.07	n.d.	3.61±0.03	5.42±0.07	0.62±0.04	0.17±0.01	1.13±0.02	29.85±0.12	9.50±0.07	302.00±.28.01	2537.50±2.12	4.21±0.03	33.15±0.09	14.41±0.07

n.d.= not detected.

Table S2. Questionnaire questions and the associated variables used in R scripts.

Questionnaire	Variables in R scripts	Comment
Chronological information	dttm	
Your id	id	
Age range	age	
Gender	gender	
Height	height	
Weight	weight	
	bmi	automatically computed combining height and weight
	body_type	automatically computed combining height and weight
knowledge of wine	oeno_exp	
knowledge of music	music_exp	
Positive surprise	wine?_pos_sur	wine? means that the same questions are repeated for each wine. The "?" charater is then replaced by the specific letter (e.g. "wineA", "wineB", etc.)
Joy / empathy / happiness	wine?_pos1	
Candor / trust / optimism / reliability	wine?_pos2	
Would you like to note a couple of positive emotions / sensations that the wine elicited in you?	wine?_pos_ann	
Negative surprise	wine?_neg_sur	
Annoyance / disgust / irritation	wine?_neg1	
Lack / weakness / incompleteness	wine?_neg2	
Would you like to note a couple of negative emotions / sensations that the wine elicited in you?	wine?_neg_ann	
How good do you find wine A when tasting it without music?	wine?_hedo	
How good do you find wine A when accompanied by the first song?	wine?_hedo_mel	
How good do you find wine A when accompanied by the second song?	wine?_hedo_upb	

Table S3. Tasting order of the different wine during the 5 events.

Event	Wine	Order
1	wineA	1
1	wineB	3
1	wineC	4
2	wineD	1
2	wineE	2
2	wineF	3
3	wineG	1
3	wineH	3
3	wineI	4
4	wineL	1
4	wineM	2
4	wineN	4
5	wineO	2
5	wineP	3
5	wineQ	4
1	wineX	2
2	wineX	4
3	wineX	2
4	wineX	3
5	wineX	1
